# Socio-economic inequalities in the consumption of fruits and vegetables in Peru between 2014 and 2019

**DOI:** 10.1017/S1368980022001860

**Published:** 2022-12

**Authors:** Akram Hernández-Vásquez, Fabriccio J Visconti Lopez, Rodrigo Vargas-Fernández

**Affiliations:** 1 Universidad San Ignacio de Loyola, Vicerrectorado de Investigación, Centro de Excelencia en Investigaciones Económicas y Sociales en Salud, 550 La Fontana Av., La Molina, Lima 15024, Peru; 2 Universidad Peruana de Ciencias Aplicadas, Lima, Peru; 3 Universidad Científica del Sur, Lima, Peru

**Keywords:** Fruit, Vegetables, Social inequalities, Latin America, Peru

## Abstract

**Objective:**

To estimate the prevalence and socio-economic inequalities in adequate consumption of fruits and vegetables in Peru between 2014 and 2019.

**Design:**

Analytical cross-sectional study. The outcome variable was adequate consumption of fruits and vegetables, defined as the consumption of five or more servings of fruits and vegetables per d (yes/no). We used concentration curves and Erreygers concentration index to describe socio-economic inequalities and a microeconometric approach to determine the contribution of each variable to inequality.

**Setting:**

Peru.

**Participants:**

Data from Peruvians aged 18 years or older collected by the Demographic and Family Health Survey.

**Results:**

The prevalence of adequate fruit and vegetable consumption did not change between 2014 (10·7 %; 95 % CI (10·0, 11·4)) and 2019 (11 %; 95 % CI (10·4, 11·7)). We found socio-economic inequalities in the adequate consumption of fruits and vegetables, with wealthier individuals having a higher prevalence of adequate consumption compared to poorer individuals in 2014 (19·2 % *v*. 3·5 %) and 2019 (18·6 % *v*. 4·7 %). The decomposition analysis found that education, urban areas and being wealthy were the main factors associated with socio-economic inequality in adequate fruit and vegetable consumption, being structural problems of society.

**Conclusion:**

Despite the current regulations on healthy eating in Peru, adequate consumption of fruits and vegetables remains low, and there are socio-economic inequalities between the poorest and wealthiest individuals. Our findings suggest that more efforts are needed to increase the intake and assess the disparities in adequate fruit and vegetable consumption.

Adequate consumption of fruits and vegetables, defined by the WHO as a daily minimum consumption of 400 g or the equivalent of five servings of fruits and vegetables a day has been associated with less mortality and disability by any cause^(1–3)^.

In addition, adequate consumption prevents some non-communicable diseases (NCD), such as CVD and cancer^(1,4,5)^, which are two of the main causes of premature mortality worldwide^(6)^. Although serving recommendations for fruit and vegetable consumption vary from country to country, most countries follow the WHO definition^(5)^. Therefore, public health interventions that improve daily consumption of fruits and vegetables represent a global health strategy for the prevention of these diseases^(5)^. However, people residing in low- and middle-income countries are less likely to follow these recommendations, because they may have a limited budget to purchase fruits and vegetables (availability and affordability) and may present a greater preference for consumption of calorie-dense foods (added sugars or ultra-processed) because of their low cost, among others^(7–10)^.

Previous research has shown that adequate consumption of fruits and vegetables can be one of the factors that contribute to social inequalities in health^(11,12)^. Factors that influence this consumption in the population have been reported at the individual (preferences of each person), local (psychosocial, sociodemographic and biological) and macro levels^(13)^. Macro-level factors, such as food policies, agriculture, distribution and production costs, are greater contributors to this consumption^(14)^. Furthermore, these factors also influence the distribution of wealth and income within societies and the private resources available to people. Consequently, they also influence disease trends and health behaviour as well as diet quality^(15)^.

Currently, according to the WHO, the average consumption of fruits and vegetables in the region of Latin America and the Caribbean (LAC) is lower than recommended, but this trend in consumption has decreased in recent years^(16)^. The factors that can hinder the consumption of fruits and vegetables in LAC are multiple, including the preference for junk food, the difficulty in its preparation, the perception that they do not satisfy hunger, the lack of publicity in the media, the cost of these foods and the lack of public policies that stimulate their consumption, among others^(17)^. The production of fruits and vegetables in Peru is widely variable among the regions and in the last years has experienced an increase in agroindustry exports that could modify the availability and consumption of domestic fruits and vegetables^(18)^. According to the 2019 Demographic and Family Health Survey (ENDES for its acronym in Spanish), only 11·3 % of the general Peruvian population consumed at least five servings of fruits or vegetables per d in that year^(19)^. In this context of low consumption of healthy foods and high rates of nutritional diseases in the population, the Healthy Eating Law was enacted in 2013 and applied in March 2019^(20)^. The objectives of this law are the promotion of healthy dining rooms in educational institutions and hospitals, and front-of-package warning labels on unhealthy foods^(18,21)^.

In LAC, 70 % of all deaths and 60 % of the burden of disease in the region are due to NCD, such as CVD, cancers, diabetes and obesity. This burden of disease can be exacerbated due to the low frequency of adequate fruit and vegetable consumption reported in Peru, especially among low-income people. However, to date, there is no evidence showing socio-economic inequalities related to adequate consumption of fruits and vegetables in Peru. Likewise, the importance of the study of disease burden for public policies in Peru and other countries is relevant, because it would evaluate the impact of a public policy (Healthy Eating Law) on the general population. This study aims to estimate the prevalence and socio-economic inequalities in the adequate consumption of fruits and vegetables in Peru between 2014 and 2019.

## Methods

### Data

ENDES is a survey containing nationally representative data on the health of Peruvians aged 15 years or older. ENDES is carried out every year by the National Institute of Statistics and Informatics (INEI for its acronym in Spanish) in Peru using a two-stage, probabilistic, stratified and balanced sampling, and its results are representative at the urban/rural and department levels. The primary sampling unit is composed of the clusters selected by probability proportional to their size. The secondary sampling unit is composed of the dwellings selected by balanced sampling. Further methodological details of the survey can be found elsewhere^(22)^.

### Sample size

We included two rounds of the ENDES survey (2014 and 2019) and the samples included individuals who were aged 18 years at the time of the survey administration. The rounds were selected because they are the oldest and the newest surveys that include information about the outcome variable. ENDES 2014 and ENDES 2019 interviewed 27 633 and 33 396 households, respectively, but after excluding pregnant women (ENDES 2014: 397; ENDES 2019: 490) and missing values (ENDES 2014: 38), the final sample size consisted of 25 485 (ENDES 2014) and 31 035 (ENDES 2019) individuals.

### Outcome variable

The outcome of interest was self-reported adequate consumption of fruits and vegetables. Participants were asked about eating habits over the 7 d prior to the survey. We considered consumption adequate if an individual consumed five or more servings of fruits and vegetables per d and inadequate when less than five or more servings of fruits and vegetables per d were consumed. The threshold used to define adequate fruit and vegetable consumption is in line with the WHO recommendation to consume a daily minimum of 400 g of fruit and vegetables, or the equivalent of five portions of fruit and vegetables per d^(5)^. In addition, this threshold has been used in previous studies^(1,3)^.

### Socio-economic variable

The socio-economic variable was the wealth index. This index is built based on a measure of household wealth constructed using the principal component analysis method which considers availability of goods, services and housing characteristics^(23)^. A higher index denotes higher wealth. Respondents were ranked according to the wealth index and were assigned to quintiles of wealth (poorest, poorer, middle, wealth and wealthiest).

### Independent variables

We included the following independent variables in the analysis: sex (male/female), age in years (18–29 years/30–59 years/60 years or more), marital status (single/married or cohabiting/separated, divorced or widowed), education level (no formal school/primary/secondary/higher), area (urban/rural), altitude measured in metres above the sea level (masl) (0–499 masl/500–1499 masl/1500–2999 masl/3000 masl or more) and the natural regions (Jungle/Highlands/Rest of the coast/Metropolitan Lima), as used in other studies^(1,3,24)^.

### Statistical analysis

To describe the population characteristics and the prevalence of the adequate consumption of fruits and vegetables, we used weighted frequencies together with their 95 % confidence interval (95 % CI). The chi-square test was used to compare the prevalence across subgroups.

To describe the socio-economic inequalities in the outcome of interest, we used concentration curves (CC) and the concentration index. The CC is a graph that plots the cumulative percentage of the outcome of interest of the population on the *y*-axis and compares it with the cumulative percentage of the population (*x*-axis), ranked according to the wealth index and starting with the poorest individuals and ending with the wealthiest individuals. The line of equity in the CC is defined as a 45-degree line plotted from the bottom left-hand corner to the upper right-hand corner. If the outcome of interest takes higher (lower) values among poorer individuals, then the CC will lie above (below) the line of equity. However, if, irrespective of their socio-economic status, individuals have exactly the same value of the outcome of interest, then the CC will be the same line of equity. The farther the CC is above the line of equality, the more concentrated the outcome of interest is among the poor individuals, or the farther the CC is below the line of equity, the more concentrated the outcome of interest is among the wealthiest individuals^(25)^.

On the other hand, the concentration index is a relative measure of inequality defined as twice the area between the CC and the line of equity. In other words, the concentration index can be defined as 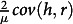 , where *μ* represents the mean of the outcome of interest, *h* denotes the outcome of interest and *r* is the cumulative percentage that each individual represents over the total population after ranking the outcome of interest by the wealth index^(25)^. The concentration index ranges from *μ* – 1 and 1 – *μ* ^(26)^. If the CC lies above (below) the line of equity, then the concentration index takes a negative/positive value. The higher the absolute value of the concentration index, the higher the magnitude of socio-economic inequality. As the outcome of interest is a dichotomous variable (yes/no), we performed the Erreygers concentration index (ECI) using the ‘*conindex*’ command in Stata^(27)^. For dichotomous variables, ECI has methodological advantages in comparison with the standard concentration index, since it considers the bounded nature of the variable^(27)^. ECI can be defined as 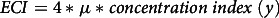 , where *y* is the outcome of interest.

To determine the contribution of each variable to the inequality in the outcome of interest, we decomposed the ECI using generalised linear models (GLM). In comparison with other approaches such as the ordinary least squares or probit estimations, generalised linear model has demonstrated to be the best choice when decomposing a dichotomous variable^(28)^. The outcome of interest 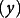 can be modelled as follows:

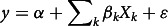

where 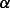 is an intercept, 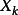 represents the independent variables previously described and that predicts 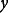 , 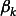 is the coefficient of 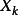 , and 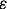 is the stochastic term of error. By running this model, ECI can be decomposed as follows:

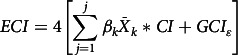

where 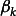 is the partial effect of the outcome of interest, 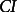 is the concentration index of 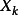 and is the generalised concentration index of the stochastic term of error. According to the model, an independent variable contributes to inequality in the outcome of interest when it is correlated with the outcome of interest, and it is unequally distributed across the wealth index. The higher the partial effect of an independent variable, and the more unequally the variable is distributed with respect to the wealth index, the higher the contribution of that variable to inequality^(25)^.

The econometric decomposition brings elasticity, concentration, the contribution and the percentage of contribution to the inequality for each independent variable. The elasticity denotes the change in the outcome of interest associated with a one-unit change in the independent variable. A positive and negative sign in elasticity indicates an increasing or decreasing change in the outcome of interest in association with a positive change in the independent variable^(25)^. The concentration index represents the concentration index of the independent variables with reference to the wealth index. A positive/negative value means that the outcome of interest is more frequent among the wealthiest/poorest individuals^(25)^. Lastly, the contribution and percentage contribution represents the absolute and relative contribution of each independent variable included in the model to the overall socio-economic-related inequality in the outcome of interest. A positive/negative contribution or percentage contribution in a variable results in an increase/decrease in the observed socio-economic inequality^(25)^.

All the analyses were performed including the expansion factor and ENDES sample specifications and were carried out using Stata v14.2 (StataCorp., 2016).

### Ethics statement

This study did not require the approval of an institutional ethics committee, since the databases are fully anonymised and are freely and publicly available from the website of INEI (https://www.inei.gob.pe/).

## Results

The description of the study sample for both periods of the ENDES survey is reported in Table 1. For both years (2014 and 2019), the sample comprised slightly more women than men and most of the individuals were aged between 30 and 59 years old. Married or cohabiting was the most frequent marital status reported by individuals, and the majority had secondary as an educational level. Most of the individuals lived in urban areas and between 0 and 499 metres above sea level (masl).

**Table 1:** Description of the study sample

Characteristics	2014			2019		
	*n*	Weighted %	95 % CI	*n*	Weighted %	95 % CI
Sample size	25 485	100·0		31 035	100·0	
Sex						
Male	11 900	46·6	45·7, 47·6	13 315	48·6	47·7, 49·5
Female	13 585	53·4	52·4, 54·3	17 720	51·4	50·5, 52·3
Age groups, years						
18–29	6115	27·5	26·6, 28·4	8548	26·7	25·9, 27·5
30–59	13 867	53·8	52·9, 54·7	17 305	54·6	53·7, 55·5
60 or more	5503	18·7	17·9, 19·5	5182	18·7	18·0, 19·4
Marital status						
Single	4775	24·0	23·1, 24·8	4229	16·8	16·1, 17·5
Married or cohabiting	15 909	62·2	61·3, 63·0	21 493	66·0	65·1, 66·9
Separated/divorced/widowed	4801	13·9	13·3, 14·5	5313	17·2	16·5, 17·9
Education level						
No formal school	1969	5·5	5·1, 6·0	1677	4·0	3·7, 4·3
Primary	7866	25·8	24·9, 26·6	7883	20·7	20·1, 21·4
Secondary	9213	39·3	38·2, 40·5	12 208	39·5	38·6, 40·5
Higher	6437	29·4	28·2, 30·6	9267	35·8	34·8, 36·8
Wealth index						
Poorest	7458	18·3	17·3, 19·3	9929	18·1	17·5, 18·7
Poorer	6239	19·0	17·8, 20·3	7883	21·0	20·2, 21·9
Middle	4759	19·9	18·9, 20·8	5623	20·6	19·8, 21·4
Richer	3796	21·1	20·0, 22·2	4293	20·1	19·3, 21·0
Richest	3233	21·7	20·3, 23·2	3307	20·1	19·2, 21·0
Natural regions						
Jungle	5052	12·1	11·1, 13·2	7113	11·8	11·1, 12·4
Highlands	10 532	30·2	28·6, 31·9	11 583	24·4	23·4, 25·3
Rest of the Coast	6876	25·9	24·1, 27·7	8760	25·4	24·6, 26·3
Metropolitan Lima	3025	31·7	30·0, 33·6	3579	38·4	37·4, 39·5
Area						
Rural	9796	24·5	23·7, 25·4	10 897	18·9	18·4, 19·4
Urban	15 689	75·5	74·6, 76·3	20 138	81·1	80·6, 81·6
Altitude (masl)						
0–499	12 254	62·5	60·7, 64·2	15 058	66·5	65·3, 67·8
500–1499	2735	7·8	6·5, 9·4	3611	7·9	6·9, 8·9
1500–2999	3632	11·5	10·3, 12·8	4800	11·4	10·6, 12·2
3000 or more	6864	18·2	16·7, 19·7	7566	14·2	13·4, 15·1

CI, confidence interval; masl, metres above sea level.

Estimates include the weights and ENDES sample specifications.

Table 2 summarises the prevalence of adequate fruit and vegetable consumption. There was no statistical difference in the prevalence of adequate fruit and vegetable consumption between 2014 (10·7 % (95 % CI (10·0, 11·4)) and 2019 (11 % (95 % CI (10·4, 11·7)). The prevalence of adequate consumption of fruits and vegetables was higher among women for both years (*P*-value = 0·004 and *P*-value = 0·002 for 2014 and 2019, respectively) as well among more educated and among the wealthiest individuals who showed a higher consumption of fruits and vegetables for both years. In addition, individuals living in an urban area (12·7 % in 2014 and 12·3 % in 2019), living in the Metropolitan Lima (14 % in 2014 and 13 % in 2019), or living between 0 and 499 masl (13·6 % in 2014 and 12·4 % in 2019) reported a higher consumption of fruits and vegetables in comparison with those living in rural areas (4·4 % in 2014 and 5·4 % in 2019), living in another natural region of Peru (Highlands: 5·2 % in 2014 and 7·5 % in 2019; rest of Coast: 13·6 % in 2014 and 11·9 % in 2019; and Jungle: 9·6 % in 2014 and 10·1 % in 2019) or living at a higher altitude (500–1499: 9·2 % in 2014 and 10·9 % in 2019; 1500–2999: 7·3 % in 2014 and 11·4 % in 2019; and 3000 or more: 3·7 % in 2014 and 4·4 % in 2019), respectively. There were no differences in adequate consumption of fruits and vegetables according to age (*P*-value = 0·130 and *P*-value = 0·174 for 2014 and 2019, respectively).

**Table 2:** Prevalence of consumption of ≥5 servings of fruits and vegetables per d in the general population and subgroups of population between 2014 and 2019

Characteristics	2014					2019				
	≥5 servings of fruits and vegetables per d					≥5 servings of fruits and vegetables per d				
	No		Yes		*P*-value*	No		Yes		*P*-value*
	Weighted %	95 % CI	Weighted %	95 % CI		Weighted %	95 % CI	Weighted %	95 % CI	
Sample size	89·3	88·6, 90·0	10·7	10·0, 11·4		89·0	88·3, 89·6	11·0	10·4, 11·7	
Sex										
Male	90·2	89·3, 91·0	9·8	9·0, 10·7	0·004	89·9	89·0, 90·8	10·1	9·2, 11·0	0·002
Female	88·5	87·5, 89·4	11·5	10·6, 12·5		88·1	87·2, 88·9	11·9	11·1, 12·8	
Age groups, years										
18–29	88·3	86·9, 89·5	11·7	10·5, 13·1	0·130	88·9	87·8, 90·0	11·1	10·0, 12·2	0·174
30–59	89·6	88·7, 90·4	10·4	9·6, 11·3		88·6	87·7, 89·4	11·4	10·6, 12·3	
60 or more	90·0	88·4, 91·4	10·0	8·6, 11·6		90·2	88·5, 91·6	9·8	8·4, 11·5	
Marital status										
Single	87·1	85·6, 88·5	12·9	11·5, 14·4	<0·001	87·4	85·7, 89·0	12·6	11·0, 14·3	0·057
Married/cohabiting	90·0	89·2, 90·8	10·0	9·2, 10·8		89·2	88·4, 89·9	10·8	10·1, 11·6	
Separated/divorced/widowed	89·7	88·2, 91·1	10·3	8·9, 11·8		89·8	88·3, 91·1	10·2	8·9, 11·7	
Education level										
No formal school	97·5	96·0, 98·5	2·5	1·5, 4·0	<0·001	97·5	95·9, 98·4	2·5	1·6, 4·1	<0·001
Primary	93·8	92·9, 94·7	6·2	5·3, 7·1		93·8	92·7, 94·6	6·2	5·4, 7·3	
Secondary	89·1	88·1, 90·1	10·9	9·9, 11·9		90·5	89·6, 91·4	9·5	8·6, 10·4	
Higher	84·0	82·4, 85·4	16·0	14·6, 17·6		83·6	82·3, 84·8	16·4	15·2, 17·7	
Wealth index										
Poorest	96·5	95·8, 97·2	3·5	2·8, 4·2	<0·001	95·3	94·6, 96·0	4·7	4·0, 5·4	<0·001
Poorer	93·3	92·3, 94·2	6·7	5·8, 7·7		91·4	90·4, 92·3	8·6	7·7, 9·6	
Middle	90·0	88·7, 91·1	10·0	8·9, 11·3		89·4	88·0, 90·7	10·6	9·3, 12·0	
Wealthier	87·5	85·9, 88·9	12·5	11·1, 14·1		87·9	86·3, 89·3	12·1	10·7, 13·7	
Wealthiest	80·8	78·7, 82·7	19·2	17·3, 21·3		81·4	79·5, 83·2	18·6	16·8, 20·5	
Natural regions										
Jungle	90·4	89·1, 91·6	9·6	8·4, 10·9	<0·001	89·9	88·9, 90·8	10·1	9·2, 11·1	<0·001
Highlands	94·8	94·2, 95·4	5·2	4·6, 5·8		92·5	91·7, 93·3	7·5	6·7, 8·3	
Rest of the Coast	86·4	84·9, 87·7	13·6	12·3, 15·1		88·1	87·2, 89·0	11·9	11·0, 12·8	
Metropolitan Lima	86·0	84·2, 87·6	14·0	12·4, 15·8		87·0	85·5, 88·5	13·0	11·5, 14·5	
Area										
Rural	95·6	94·9, 96·2	4·4	3·8, 5·1	<0·001	94·6	93·9, 95·3	5·4	4·7, 6·1	<0·001
Urban	87·3	86·3, 88·1	12·7	11·9, 13·7		87·7	86·8, 88·4	12·3	11·6, 13·2	
Altitude (masl)										
0–499	86·4	85·4, 87·4	13·6	12·6, 14·6	<0·001	87·6	86·7, 88·5	12·4	11·5, 13·3	<0·001
500–1499	90·8	89·0, 92·4	9·2	7·6, 11·0		89·1	87·3, 90·6	10·9	9·4, 12·7	
1500–2999	92·7	91·5, 93·7	7·3	6·3, 8·5		88·6	87·1, 89·9	11·4	10·1, 12·9	
3000 or more	96·3	95·5, 97·0	3·7	3·0, 4·5		95·6	94·8, 96·2	4·4	3·8, 5·2	

CI, confidence interval; masl, metres above sea level.

Estimates include the weights and ENDES sample specifications.

* *P*-value for *χ* ^2^ test.

**Fig. 1:**
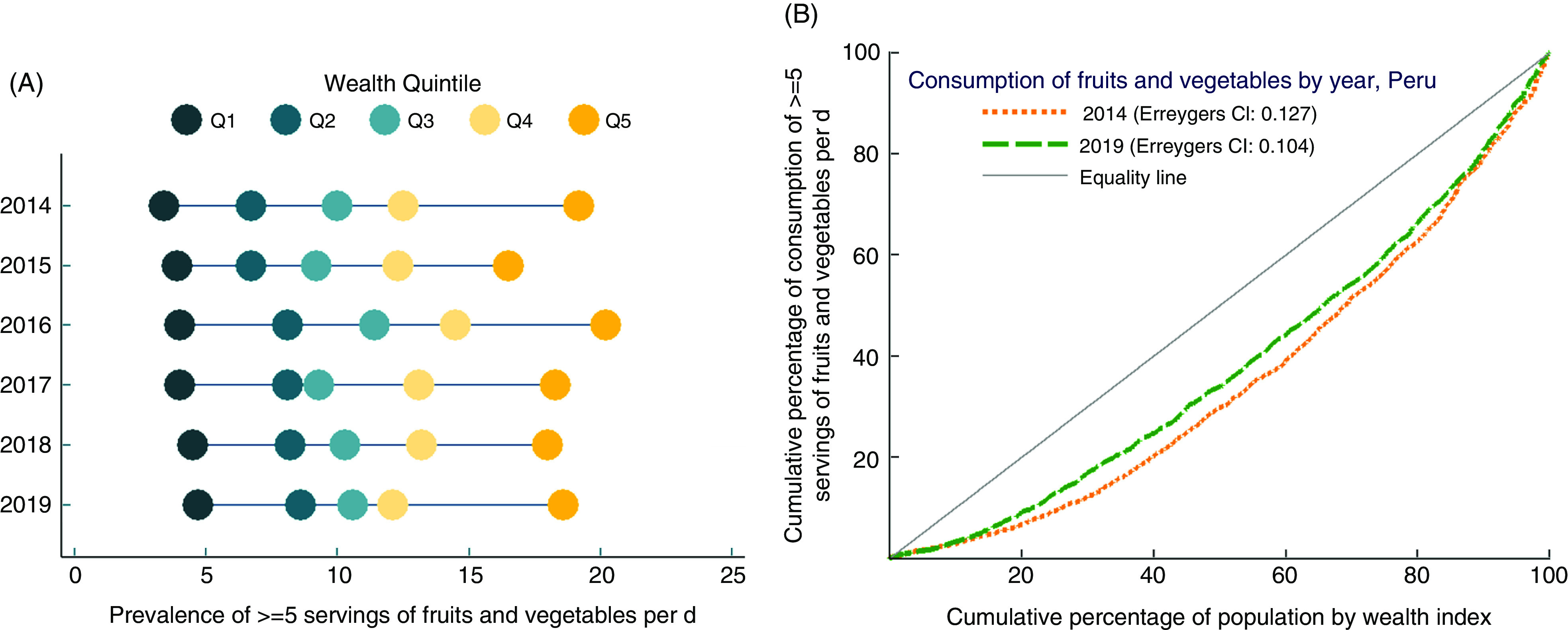
Panel A. Equiplot of inequality of ≥5 servings of fruits and vegetables per d. Panel B. Concentration curves for inequality of ≥5 servings of fruits and vegetables per d between 2014 and 2019. Panel A was constructed using data from ENDES 2014, 2015, 2016, 2017, 2018 and 2019

Panel A of Fig. 1 shows the prevalence of adequate consumption of fruits and vegetables according to the wealth quintiles and between the 2014 and 2019 time frame. In both years (2014 and 2019), the gap between the wealthiest and the poorest in adequate fruit and vegetable consumption remained at nearly 15 % points. Panel B of Fig. 1 shows that for both years, the CC was far below the line of equity, meaning that the consumption of fruits and vegetables was concentrated among the wealthiest individuals. Despite the ECI being lower in 2019 (ECI = 0·104) in comparison with 2014 (ECI = 0·127), we found no difference in the CC for both years after running the dominance test with the intersection union principle. This result indicates that there were no differences in the inequality of fruit and vegetable consumption between 2014 and 2019.

Table 3 reports the decomposition analysis of the outcome of interest for 2014 and 2019. The elasticity value for higher education was 0·134 and 0·198 for 2014 and 2019, respectively, indicating that a 1 % change in individuals from no formal school to higher education may result in a 13 % and 19 % change in the increasing of the inequality in the adequate consumption of fruits and vegetables. On the other hand, the elasticity value for the natural region showed that for a 1 % change in individuals living from Jungle to the Metropolitan Lima is associated with a 4·6 % decrease in the inequality in the adequate consumption of fruits and vegetables. For the concentration index, adequate fruit and vegetable consumption in individuals with a secondary or higher education, individuals living in the Metropolitan Lima or individuals living in the urban area was concentrated among the wealthiest individuals. Lastly, we found that higher education, urban areas and the wealthiest individuals are the main factors associated with socio-economic inequality in the adequate consumption of fruits and vegetables.

**Table 3:** Decomposition of the concentration index of consumption of ≥5 servings of fruits and vegetables per d between 2014 and 2019

Predictors	2014				2019			
	Elasticity	CI	Contribution	%	Elasticity	CI	Contribution	%
Sex								
Male	Reference	Reference	Reference	Reference	Reference	Reference	Reference	Reference
Female	0·047	0·016	0·001	0·6	0·050	0·017	0·001	0·8
Age groups, years								
18–29	Reference	Reference	Reference	Reference	Reference	Reference	Reference	Reference
30–59	−0·010	0·011	0·000	−0·1	0·012	0·055	0·001	0·7
60 or more	0·007	0·004	0·000	0·0	0·008	0·018	0·000	0·1
Marital status								
Single	Reference	Reference	Reference	Reference	Reference	Reference	Reference	Reference
Married or cohabiting	−0·024	−0·106	0·003	2·0	−0·015	−0·037	0·001	0·5
Separated/divorced/widowed	−0·005	−0·004	0·000	0·0	−0·007	−0·015	0·000	0·1
Education level								
No formal school	Reference	Reference	Reference	Reference	Reference	Reference	Reference	Reference
Primary	0·067	−0·371	−0·025	−19·5	0·057	−0·317	−0·018	−17·2
Secondary	0·149	0·020	0·003	2·4	0·155	−0·117	−0·018	−17·4
Higher	0·134	0·459	0·061	48·5	0·198	0·524	0·104	99·5
Wealth index								
Poorest	Reference	Reference	Reference	Reference	Reference	Reference	Reference	Reference
Poorer	0·020	−0·338	−0·007	−5·4	0·024	−0·360	−0·009	−8·3
Middle	0·038	−0·044	−0·002	−1·3	0·031	−0·009	0·000	−0·3
Richer	0·057	0·300	0·017	13·5	0·035	0·319	0·011	10·6
Richest	0·095	0·680	0·064	50·8	0·066	0·643	0·043	41·0
Natural regions								
Jungle	Reference	Reference	Reference	Reference	Reference	Reference	Reference	Reference
Highlands	−0·015	−0·388	0·006	4·6	0·008	−0·342	−0·003	−2·6
Rest of the Coast	−0·013	0·081	−0·001	−0·8	−0·015	0·036	−0·001	−0·5
Metropolitan Lima	−0·046	0·523	−0·024	−18·9	−0·045	0·509	−0·023	−21·9
Area								
Rural	Reference	Reference	Reference	Reference	Reference	Reference	Reference	Reference
Urban	0·019	0·655	0·013	10·0	0·033	0·548	0·018	17·3
Altitude (masl)								
0–499	Reference	Reference	Reference	Reference	Reference	Reference	Reference	Reference
500–1499	−0·005	−0·081	0·000	0·3	0·001	−0·071	0·000	−0·1
1500–2999	−0·021	−0·085	0·002	1·4	−0·006	−0·100	0·001	0·6
3000 or more	−0·062	−0·296	0·019	14·6	−0·050	−0·255	0·013	12·3
Residual			−0·003				−0·016	

CI, confidence interval; masl, metres above sea level.

Estimates included the weights and ENDES sample specifications.

## Discussion

The present study identified socio-economic inequalities in fruits and vegetables consumption in the Peruvian population between 2014 and 2019. We found socio-economic inequality in adequate consumption of fruits and vegetables, and the gap of adequate consumption between richest and poorest remained constant between 2014 and 2019. In addition, the decomposition analysis of inequalities showed that having a higher education, belonging to the highest wealth quintiles and residing in urban areas were the variables with the highest positive contributions to this inequality. Strategies and social programmes have been implemented in Peru such as ‘Guías Alimentarias para la Población Peruana’, ‘Alimentación y Nutrición Saludable’, ‘Juntos’, ‘Qali Warma’, ‘Haku Wiñay’, ‘Proyecto Educativo Nutricional Pachacútec’ and ‘Vaso de Leche’ to improve the nutritional status of the Peruvian population by providing access to safe food and reducing the prevalence and morbidity and mortality from nutritional-related diseases, especially in vulnerable groups and in people living in extreme poverty^(29–31)^. However, our results show that it is probably difficult for regional governments to implement and scale up these policies, in addition to economic limitations in rural areas, low agricultural production due to poor training of farmers and territorial characteristics affected by climate change^(30,31)^. Therefore, due to the difficulty to adequately implement the above-mentioned policies and programmes, the socio-economic inequality in adequate fruits and vegetables consumption is maintained, which may contribute to a high burden of disease as a consequence of nutritional deficit, especially in people living in poverty and living in rural areas^(32)^.

The prevalence of adequate fruits and vegetables consumption per d showed no significant change between 2014 (10·7 %) and 2019 (11·0 %). Our estimates are lower than those reported for 28 low- and middle-income countries (18 %; 95 % CI (16·6, 19·4)), although they are higher than the prevalence described for LAC (8 %; 95 % CI (7·4, 8·6))^(33)^. Our results show that the consumption of fruits and vegetables in the Peruvian population is low compared to other low- and middle-income countries. This highlights the need to develop effective policies in Peru and strengthen current strategies to promote the consumption of healthy foods such as fruits and vegetables by focusing on training farmers, increasing agricultural production, reducing the cost of fruits and vegetables and improving the implementation of these policies in rural areas. These strategies may help Peru to meet with the Sustainable Development Goals by 2030, considering that adequate consumption of fruits and vegetables is included within the second and third Sustainable Development Goals that highlight the need for consumption of adequate amounts of fruits and vegetables per d as part of a diversified and healthy diet.

We found a concentration of adequate fruits and vegetables consumption per d among people with a higher wealth index. Similar findings have been described in other studies conducted worldwide^(24,34–37)^ that report that more people with a high socio-economic level have an adequate consumption of fruits and vegetables compared to the poorest. An explanation for this could be that people living in lower-income households make less healthy and lower-quality food purchases and have less access to fruits and vegetables due to the high cost of this type of food^(38)^. In addition, some barriers related to access to and the availability of healthy foods, low knowledge about a balanced diet, residing in rural areas and cultural habits of poor nutrition could contribute to inadequate fruits and vegetables consumption in lower-income households^(31,39)^. Therefore, in the Peruvian context, food assistance programs could be a key intervention target to improve fruit and vegetable consumption. The effectiveness of these programmes in low-income households has been evaluated in randomised community-based studies conducted in the USA, in which interventions focused on restricting consumption of calorie-dense foods and providing economic incentives for the purchase of fruits and vegetables increased fruit and vegetable consumption and decreased expenditures on ultra-processed foods. Although these strategies were implemented in a context different from that of Peru, their findings could be useful to design similar strategies in the Peruvian population living in poverty and be implemented together with existing social programmes such as ‘Juntos’, which provides conditional cash transfers to individuals, to improve the impact of this programme and mitigate food insecurity and its consequences for health^(40,41)^.

In addition, the decomposition analysis revealed that having a higher education, belonging to the highest wealth quintiles and residing in an urban area are the main factors associated with socio-economic inequalities for adequate consumption of fruits and vegetables, being structural problems of society. A higher education level was the largest contributor to inequalities between 2014 and 2019 (with an increase of 51 % in 2019), which is similar to what was reported in a previous study conducted in South Africa^(42)^, in which educational level was the largest contributor to inequalities in fruit and vegetable consumption in the population. In Peru, a study conducted in community kitchens (places that provide food to low-income people) in Metropolitan Lima in 2013 found that people with more than 12 years of education had a fourfold higher fruit and vegetable consumption than people with no education^(39)^. Based on the literature, a higher education level is related to a higher consumption of fruits and vegetables due to a greater knowledge of food and nutritional information and the health benefits of fruits and vegetables and financial availability to purchase fruits and vegetables^(43,44)^.

On the other hand, the highest household welfare quintiles contributed to more than 40 % of the inequalities in fruit and vegetable consumption between 2014 and 2019 (with a decrease of 9·1 percentage points in 2019), while an urban place of residence contributed 10 % of the inequalities in 2014 and 17·3 % in 2019. These results are similar to those reported in previous studies conducted in Iran and India^(36)^. The contribution of inequalities in the highest welfare quintiles can be attributed to household purchasing power, in which the cost of two servings of fruit and three servings of vegetables per d represents more than 50 % of household income in low-income countries, which may reflect the unaffordability of lower-income households to purchase fruit and vegetables^(45)^. In addition, people with lower incomes face situations of insecurity related to employment, food and housing, and activate psychological and behavioural mechanisms that influence consumption and dietary choice^(46)^. Lastly, the contribution of the urban residence could be associated with the problems existing in rural areas, such as the distance of rural households to the nearest supermarket, the omission or loss of meals, inability to consume balanced meals in rural households, and lack of knowledge about the benefits of fruit and vegetable consumption^(35)^. Therefore, strategies to increase fruits and vegetables consumption in populations residing in rural areas and with lower socio-economic status should be implemented according to the needs and conditions of the region. It is worth noting that, in Peru, the highest poverty rate is found in the Sierra and Selva regions, which correspond to populations that reside mostly in rural areas^(47)^.

### Policy implications

Our findings may have some policy implications to note. First, in order to assess the inequalities in the consumption of fruits and vegetables, different strategies that have demonstrated to be effective elsewhere^(48)^ can be adapted to local context to strengthen the current strategies in Peru. These strategies can range from school-based interventions to parent and family home-based interventions, and from workplace-based interventions to mass media- and electronic-based interventions. Second, taxes and subsidies can be tools for improving fruit and vegetable consumption. For example, countries such as Denmark, the USA and Mexico have introduced fiscal strategies targeting food and beverages to improve public health nutrition^(49)^. In addition, evidence suggests that a reduction of 10 % of the price for fruit and vegetables is associated with an increased intake of 14 %^(49)^. To support this strategy in Peru, it is a must to ensure that fiscal measures do not exacerbate the current inequalities by increasing financial burden to the poorest individuals. Third, since the supply of fruits and vegetables in rural areas may be challenging^(50)^, to increase consumption and reduce inequalities in rural settings, it is important to improve architecture and couple this strategy with pricing strategies. All these strategies could be designed together with health strategies such as ‘Alimentación y Nutrición Saludable’ or social programmes such as ‘Juntos’ or ‘Vaso de Leche’ that have previously been implemented in Peru to improve consumption and access to fruits and vegetables and ensure safe nutrition in the Peruvian population, especially in people living in extreme poverty and in rural areas.

### Limitations and strengths

The information on the consumption of fruits and vegetables was self-reported by the respondents in the last 7 d prior to the survey, which limits the evaluation to a short period of time, and it would not be possible to establish the consumption that a person has had throughout their life. In addition, there could be a memory bias due to variables on information from the past, as well as errors in the recording of the information by the interviewer. It should also be considered that since this is a secondary data study, there is relevant information that has not been measured by the ENDES (e.g. the absolute amount of fruits and vegetables consumed, the diversity and type of fruits consumed and their quality, among others) that could allow better characterisation of this risk factor in the population. However, the ENDES is a nationally representative survey in Peru that collects annual information on fruit and vegetable consumption based on the WHO STEPwise Approach to NCD Risk Factor Surveillance (STEPS), which is a widely used tool to measure fruit and vegetable consumption and provides the data to obtain official estimates of risk factors for NCD in the Peruvian population.

## Conclusion

In conclusion, our results show that only one in ten Peruvians reported adequate consumption of fruits and vegetables. In addition, we found socio-economic inequalities in adequate consumption of fruits and vegetables, and the gap in adequate consumption between the poorest and wealthiest remained unchanged between 2014 and 2019. Despite the norms and guidelines established on healthy eating and food safety in Peru, it is necessary to strengthen the strategies for promotion and access to healthy food consumption to reduce the burden of disease and health spending on NCD, especially in vulnerable groups and in people living in rural areas and in extreme poverty.
